# Exploring the Therapeutic Potential of *Podophyllum hexandrum* Root Extract: Chemical Composition, Antimicrobial Efficacy, and Antioxidant and Anticancer Activities

**DOI:** 10.1155/sci5/5100547

**Published:** 2025-03-18

**Authors:** Mohmmad Ashaq Sofi, Mohd Abass Sofi, Anima Nanda, B. K. Nayak, Zulhabri Othman, Muhammad Zulfiqah Sadikan

**Affiliations:** ^1^Department of Biomedical Engineering, Sathyabama Institute of Science & Technology, Chennai 600119, Tamil Nadu, India; ^2^Department of Chemistry, Sathyabama Institute of Science &Technology, Chennai 600119, Tamil Nadu, India; ^3^Department of Botany, K. M. Government Institute for Postgraduate Studies and Research (Autonomous), Puducherry 605008, India; ^4^Department of Biochemistry, Faculty of Medicine, Manipal University College Malaysia, Melaka 75150, Malaysia; ^5^Department of Pharmacology, Faculty of Medicine, Manipal University College Malaysia, Melaka 75150, Malaysia

**Keywords:** antimicrobial properties, antioxidant activity, chemical composition, cytotoxicity, *P*. *hexandrum*, root extract

## Abstract

Medicinal plants have been used for centuries as therapeutic compounds to address various health issues. Their rich phytochemical composition offers diverse bioactive substances with potential health benefits. This study aimed to explore the therapeutic potential of *Podophyllum hexandrum* root extract by investigating its chemical composition and antimicrobial, antioxidant, and anticancer properties. The phytochemical profiling of *P. hexandrum* root was conducted using GC-MS analysis, which identified 26 compounds in the extract. The ethanolic root extract displayed strong inhibitory effects in the well diffusion assay against all tested microbes, with minimum inhibitory concentration (MIC) values ranging from 64 to 256 μg/mL. *Candida albicans* exhibited the lowest MIC value of 64 μg/mL. The antioxidant activity of the extracts was compared to standard antioxidant, revealing a dose-dependent response with a notable radical scavenging activity of 59.23% at 100 μg/mL. Furthermore, the extract demonstrated strong cytotoxic effects against the human cancer cell line HT-29, with IC_50_ values of 38.20 and 32.5 μg/mL for 24 and 48 h. Overall, this study emphasizes the remarkable antibacterial, antioxidant, and anticancer properties of *P. hexandrum* root extract.

## 1. Introduction

Medicinal plants have long been recognized for their potential to complement or even substitute modern medical treatments. The use of plants for therapeutic purposes is rooted in traditional medicine and has garnered increasing attention from both the scientific and medical communities [1, 2]. There is a pressing requirement to assess the therapeutic capabilities of herbal remedies to encourage their use. However, efforts to promote traditional medicine have been relatively limited, with much work remaining to be done in this regard [3]. One of the primary obstacles in global healthcare is the requirement for innovative, efficacious, and cost-effective pharmaceuticals to address microbial illnesses, particularly in developing nations. Certain species of *Staphylococcus* and *Streptococcus* bacteria are known to cause respiratory and skin infections. Additionally, *Pseudomonas,* members of *Enterobacteriaceae*, and *Candida albicans* can lead gastrointestinal urogenital illnesses and wound infections. These microorganisms often exhibit resistance to conventional antibiotics [4]. Therefore, it is crucial to assess the therapeutic efficacy of herbal medicines further [5]. The buildup of free radicals within the body can result in oxidative stress. Antioxidants are compounds capable of counteracting free radicals, binding metal ions, preventing lipid peroxidation, and exhibiting reducing characteristics. They are vital in safeguarding the human body against oxidative stress [6]. Oxidative stress is linked to various illnesses and circumstances, such as cancer, neurological ailments, cardiovascular disorders, Alzheimer's disease, and the aging process [7]. Plant-derived antioxidants offer several advantages, such as lower toxicity, greater effectiveness, and cost-efficiency, which has sparked a growing interest in natural plant-based antioxidants [8]. Secondary metabolites like phenolics and flavonoids found in plants are known for their potent ability to scavenge free radicals, making them valuable as antioxidants [9]. Numerous antioxidants derived from natural and synthetic sources have been suggested as potential therapeutic agents for various human ailments [10]. The incidence of various types of cancer significantly increases with age in human populations, particularly from the fourth to the eighth decade of life. Cancer kills nearly 6 million people globally, making it the top cause of mortality in men and women [11]. Approximately 60% of anticancer drugs originate from plant sources, such as taxol from *Taxus brevifolia* and camptothecin from *Cuscuta reflexa* [12]. Ideally, anticancer drugs should have minimal side effects, trigger apoptosis, and display selective cytotoxicity toward cancer cells, making them the preferred therapeutic option [13]. *P. hexandrum*, commonly called the Himalayan Mayapple, is a perennial plant native to the Himalayas. This herb has a history of traditional use in treating conditions like condylomata or venereal warts. Additionally, research has indicated its effectiveness in combating various cancer types, including lung, brain, bladder, breast, and non-Hodgkin's lymphoma. *P. hexandrum* is a rich source of podophyllotoxin, a lignan compound extensively used in the development of anticancer drugs. However, the species is endangered due to overharvesting and habitat loss, highlighting the need for sustainable conservation efforts alongside its therapeutic exploration. The study focused on examining the phytochemical composition and antimicrobial and anticancer properties of *P. hexandrum* root extract [14, 15]. While previous studies have explored the antimicrobial, antioxidant, and antitumor activities of *P. hexandrum* in isolation, a comprehensive evaluation of these properties together remains unexplored. This study integrates these activities to provide a holistic understanding of the plant's therapeutic potential, particularly focusing on its unique chemical profile from the Kashmir Himalayas. By investigating the interconnected roles of these activities, we aim to uncover synergistic effects and validate *P. hexandrum* as a multifunctional therapeutic agent for addressing modern health challenges.

The findings from this study could provide significant insights into the therapeutic capabilities of *P. hexandrum* and potentially lead to the development of new treatments for various infectious diseases and cancers.

## 2. Collection of Plant Material

**Figure 1:**
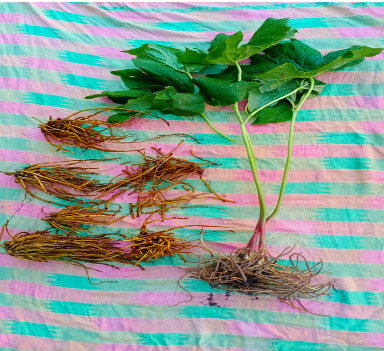
*P. hexandrum* plant.

**Figure 2:**
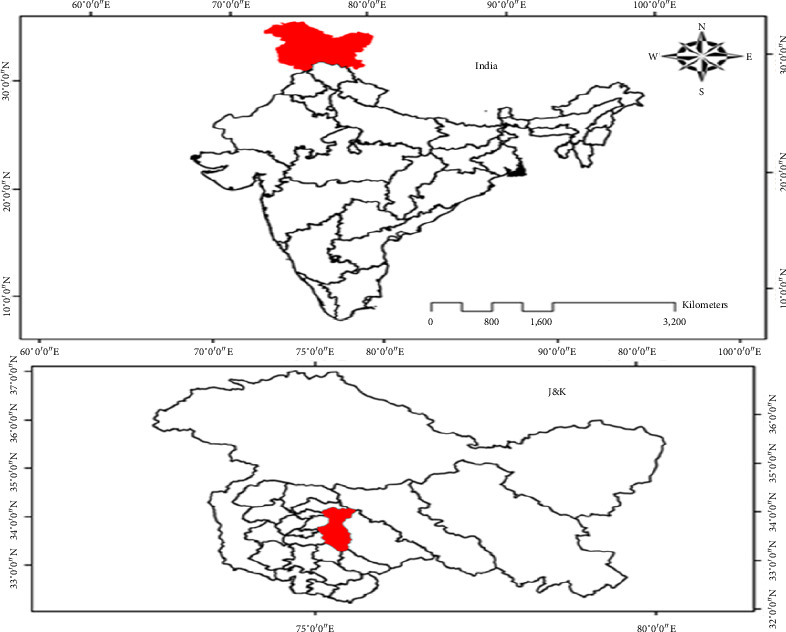
Geographical representation of the *P. hexandrum* collection site in Jammu and Kashmir, India. The plant sample was sourced from Daksum village, Anantnag district (Longitude: 75°26′6″ E, Latitude: 33°36′43″ N).

The fresh plant material of *P. hexandrum* Royle (Figure 1) was collected in June, 2022, from Daksum Village located in the Anantnag District of Jammu and Kashmir. The site is geographically positioned at Longitude 33°N 75°E, Latitude 33°36′43″ N, approximately 95 km from the capital city, Srinagar (Figure 2).To ensure the accuracy of plant identification, experts in the Centre for Plant Taxonomy at Kashmir University were consulted for biological authentication. A reference sample has been placed in the KASH-Herbarium with voucher number 3069.

### 2.1. Preparation of Extract

The plant material was subjected to extraction via a simple maceration process. 20 g of coarse plant powder was mixed with 200 mL of ethanol of the desired quality grade in a flask. The mixture was shaken for 24 h at room temperature and then filtered using Whatman No. 1 filter paper. The resulting filtrate was concentrated by a rotary evaporator (BEING RV21A) at 40°C. To ensure adequate quantity and quality, the extraction was performed three times for further analytical use.

(1) $$\begin{array}{c} \text{Total Extraction Yield }\%=\left(\frac{\text{Mass of the extract}}{\text{Mass of the sample}}\right)\times 100. \end{array}$$

### 2.2. Phytochemical Analysis

The *P. hexandrum* root extract was subjected to determine the presence of different phytochemical constituents (phenolic flavonoids, tannins alkaloids, steroids, saponins, and terpenoid compounds) using standard procedures [16].

### 2.3. Phenol Detection

1 mL of the extract was added to a test tube, followed by 1-2 drops of FeCl_3_. The presence of phenols was indicated by a color change in the solution after a few minutes.

### 2.4. Alkaloid Detection

The presence of alkaloids was determined by mixing 1 mL of the extract with 2-3 drops of Wagner reagent. A yellow coloration in the mixture served as evidence of alkaloids.

### 2.5. Flavonoid Detection

2 mL of the extract was mixed with small pieces of magnesium ribbon, and 5% concentrated HCl was added drop by drop. After 2 min, the formation of a pink-scarlet color confirmed the presence of flavonoids.

### 2.6. Terpenoid Detection

A test for terpenoids involved dissolving 1 mL of the plant extract in 1 mL of chloroform. Two drops of concentrated sulfuric acid were added, and the mixture was shaken. A yellow color appearing in the lower layer indicated terpenoids.

### 2.7. Saponin Detection

Saponins were identified by diluting 1 mL of the extract with 5 mL of distilled water in a test tube. The mixture was shaken vigorously for 15 min. The appearance of a stable foam layer on the surface indicated the presence of saponins.

### 2.8. Tannin Detection

2 mL of the plant was treated with a few drops of 10% ferric chloride solution. The development of a blue-green coloration confirmed the presence of tannins.

### 2.9. Steroid Detection

The presence of steroids was assessed by dissolving 1 mL of the extract in 1 mL of chloroform. To this solution, 1 mL of acetic anhydride and two drops of concentrated sulfuric acid were carefully added along the sides of the test tube. A red coloration in the upper layer indicated steroids.

### 2.10. GC-MS Analysis

The analysis of the compounds found in the ethanolic root extract of *P. hexandrum* was conducted using GC-MS, following a procedure previously outlined [17]. Prior to analysis, the extract was prepared in 100% ethanol. GC-MS Shimadzu QP 2010 analyzer was used to perform GC-MS. The sample was introduced into the instrument using the split mode with split ratio of 1: 2. Helium was used as carrier gas. Initially, the column oven was set to 80°C. The temperature was then gradually increased by 3°C per minute until it reached 200°C. After this, the temperature was raised to 260°C at a rate of 10°C per minute and maintained at that level for 5 min. Overall run time was 54 min. Identification of the biologically active compounds of the extract was achieved by examining their retention times and fragmentation patterns. This was done by comparing the obtained data to the NIST17.lib and WILEY8.LIB.

### 2.11. Antimicrobial Activity

The antimicrobial potential of the plant extract was assessed using well diffusion method [18]. Microbial strains, *Candia albicans* ATCC 10231*, Escherichia coli* ATCC 11229, *Pseudomonas aeruginosa* ATCC 15442, *Staphylococcus aureus* ATCC 25923, and *Enterococcus faecalis* ATCC 29212, were cultured and then evenly spread on the entire surface of MHA. Wells of 6 mm diameter were made on the agar plates and were saturated with 100 μL of extract from each stock solution of the extract prepared (5, 7.5, 10, 12.5, and 15 mg/mL). The plates were left to incubate at 37°C for 24 h. The diameter of the inhibitory zone around each well was measured. As positive controls, standard antibiotics ampilox, ciprofloxacin (30 μg), and fluconazole were employed. DMSO served as a negative control. The antimicrobial assay was conducted in triplicates.

### 2.12. Determination of Minimum Inhibitory Concentration (MIC)

The assay was carried out using a 96-well plate with flat-bottom wells, following the procedure established by Gabrielson et al. [19]. The first 10 wells of the plate were filled with varying concentrations of *P. hexandrum* root extract, ranging from 512 to 1 μg/mL. Once the wells were filled with the extract, 5 μL of 12-hour-old microbial cultures was added to each well. The plates were incubated at 37°C and left undisturbed for 16–18 h. Following the incubation, 10 μL of (5 mg/mL) MTT solution was introduced into each well. MTT is a yellow dye that living cells convert into a purple formazan product. After a 2 h incubation with MTT, 100 μL of DMSO was added to dissolve the formazan product. The visual change in color from yellow to purple was observed, and the MIC, which corresponds to the lowest concentration of the root extract that completely halted bacterial growth, was determined. This experiment was conducted in triplicate.

### 2.13. Determination of In Vitro Antioxidant Activity

The evaluation of *P. hexandrum* ethanolic extract's ability to neutralize DPPH radicals was conducted, following a method adapted from Arika et al. [20] with slight modifications. The root extract and ascorbic acid (utilized as a reference) were prepared at different concentrations, spanning from 20 to 100 μg/mL. A 1 mM DPPH solution was prepared in methanol. Subsequently, 1 mL of each dilution from the test extract and the standard reference were individually combined with 0.5 mL of the DPPH solution and 3 mL of methanol in sterile test tubes. After vigorously vortexing the mixture for 5 min, it was left in a dark environment at room temperature for 30 min. A blank solution was prepared with 3 mL of methanol and 0.5 mL of DPPH. The absorbance of the solutions was determined at 517 nm using a spectrophotometer, with the blank as a reference. The experiment was conducted in triplicate. The percentage of DPPH free radical quenching activity exhibited by the plant extracts was calculated as follows:

(2) $$\begin{array}{c} \text{DPPH }\%\text{ Inhibition}=\left(\frac{\text{Abs control}–\text{Abs sample}}{\text{Abs control}}\right)\times 100. \end{array}$$

### 2.14. ABTS + Scavenging Assay

The evaluation of *P. hexandrum* ethanolic extract's ability to neutralize ABTS + radicals was conducted, following previously adapted method [20, 21] with slight modifications. An ABTS radical cation (ABTS+) was prepared by combining 7 mM ABTS with 2.45 mM potassium persulfate in equal proportions and then allowing it to sit in a dark room at ambient temperature for a period of 12–16 h. The resultant ABTS + solution was then diluted using methanol until it achieves an absorbance of 0.700, at 734 nm. Different concentrations of the plant extract ranging from 20 to 100 μg/mL were introduced to the diluted ABTS + solution. Each combination was stirred for half an hour, after which their absorbance was noted. A solvent blank was processed for every assay. The percentage inhibition of 734 nm absorbance was computed using the formula: ABTS + neutralizing effect (%) = ((AB − AA)/AB) × 100. Here, “AB” is the absorbance of the ABTS radical mixed with methanol, while “AA” is the absorbance of the ABTS radical combined with the sample extract or standard. Ascorbic acid was utilized as the standard compound in this analysis. The experiment was conducted in triplicate.

### 2.15. Cell Culture

The HT-29 cell line was obtained from the National Centre for Cell Science (NCCS), Pune, India. The cells were cultured in Dulbecco's Modified Eagle Medium (DMEM) (Sigma-Aldrich, United States, Catalog: D1152), supplemented with 10% fetal bovine serum (FBS) (Gibco, United States, Catalog: 10270106), 100 U/mL penicillin, and 100 μg/mL streptomycin. The culture was maintained in a humidified incubator at 37°C with 5% CO_2_ to ensure optimal growth conditions. To propagate the cells, trypsinization was performed using 1X trypsin-EDTA (Gibco, United States, Catalog: 25200-056) when they reached 70%–80% confluency. The detached HT-29 cells were then seeded into a 96-well plate at an appropriate density and allowed to adhere for 21–24 h before treatment.

### 2.16. Cell Viability Assay

To assess the potential cytotoxicity of *P. hexandrum* extract, we conducted an MTT assay with modifications to a previously established protocol [22]. In brief, we seeded 6 × 103 HT-29 cells per well, enabling them to attach in a humidified environment. The HT-29 cells were then exposed to different concentrations of root extract (10–60 μg/mL) and were further incubated for 24 and 48 h. Next, we added 10 μL MTT dye (5 mg/mL) into all wells and allowed it to incubate for 4 h at 37°C. Subsequently, we dissolved the formazan crystals, which appeared purple in color, by adding 100 μL of DMSO to each well. Finally, we measured the absorbance of the formazan crystals at 590 nm using a microplate reader and calculated cell viability (%) in comparison to the untreated control. This experiment was conducted in triplicate.

(3) $$\begin{array}{c} \text{Cell viability }\%=\left(\frac{\text{absorbance of treated cells}}{\text{absorbance of control cells}}\right)\times 100. \end{array}$$

### 2.17. Statistical Analysis

The data are presented as mean ± S.D. from three independent experiments. Statistical analysis was performed using 2-way ANOVA followed by Tukey's multiple comparison test to determine significance between the control and treated groups and Sidak's multiple comparison for antioxidant assay.

## 3. Results and Discussion

The percentage of plant extract obtained using ethanol as a solvent was assessed, and the outcomes are presented in Table 1.

The phytochemical evaluation of root extract of *P. hexandrum* confirmed the presence of various phytocompounds (Table 2). The findings highlight the rich diversity of phytochemicals present in the extract.

The GC-MS chromatogram of the ethanolic root extract of *P. hexandrum* recorded a total of 29 peaks corresponding to the bioactive compounds. The phytoconstituents that were identified are listed in Table 3. The chromatogram of the *P. hexandrum* root extract clearly revealed the presence of various phytocompounds, some of which have previously been linked to antimicrobial, antioxidant, and anticancer activities.

**Figure 4:**
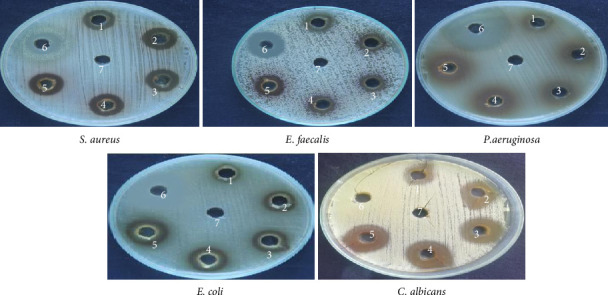
Antimicrobial activity of *P. hexandrum* ethanolic root extract against various microbial strains using the well diffusion method. Each Petri dish represents a specific microbial strain: S. aureus, E. faecalis, *P. aeruginosa*, *E. coli*, and C. *albicans*. Wells 1–5 contained 100 μL of plant extract prepared from a stock solution (5–15 mg/mL). Well 6 contained the positive control (25 μL from a 1 mg/mL stock solution of Ampilox for Gram-positive bacteria, Ciprofloxacin for Gram-negative bacteria, and Fluconazole for fungal strain). Well 7 contained the negative control DMSO.

**Figure 5:**
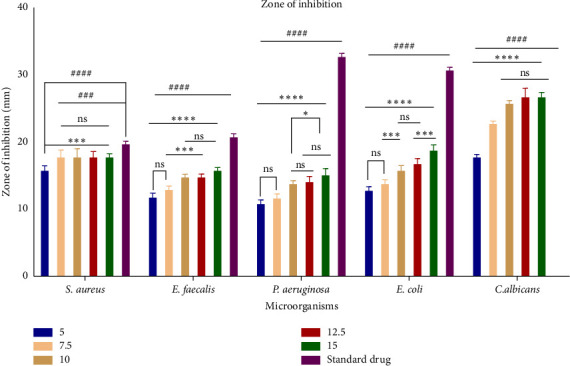
This bar chart displays the zone of inhibition (mm) of different concentrations (5, 7.5, 10, 12.5, and 15 mg/mL) of *P. hexandrum* ethanolic root extract against six different microbes: *S. aureus, E. faecalis, P. aeruginosa, E. coli,* and *C. albicans.* The data are presented as mean ± S.D. from three independent experiments and analyzed by using 2-way ANOVA followed by Tukey's multiple comparison test to determine significance (^###^*p* < 0.001 and ^####^*p* < 0.0001) between the standard and extract groups.

Among these constituents, tyrosol has been shown to possess antimicrobial, anticancer, and antioxidant activities [23, 24]. Trioxsalen, found in the ethanolic extract at a concentration of 8.08%, is a psoralen derivative and belongs to the furanocoumarin group, originating from various plants, primarily *Psoralea corylifolia*. Similar to other psoralens, it triggers photosensitization of the skin. This compound is utilized in the phototherapy treatment of conditions like vitiligo and eczema. In its photoactivated state, it induces the formation of bonds within DNA strands, ultimately leading to cellular apoptosis. Trioxsalen can also be linked to dyes for confocal microscopy, enabling the examination of DNA damage in research. It has also been investigated for its potential use in generating antisense oligonucleotides that can selectively cross-link with mutant mRNA sequences [25, 26]. 9,12-Octadecadienoic acid (Z,Z)-, n-hexadecenoic, and 9,12-octadecadienoic acid methyl ester acid have been reported to have antimicrobial activity [27]. 2,2′-Benzylidenebis(3-methylbenzofuran), a predominant compound detected in the ethanolic extract of *P. hexandrum*, has also been identified in the seeds of the *Hunteria umbellata* plant [28]. This compound showed considerable potential in forming lead targets for combating diabetes through molecular docking. 11H-Cyclopenta[a]phenanthrene-15-carboxylic acid has been reported to have antitumor activities. 3,4-Bis[(trimethylsilyl)oxy]dihydro-2(3H)-furanone is a polyphenol, a lignan, an angiogenesis inhibitor, and an antiproliferative and antiasthmatic agent [29–31]. The exploration of the antibacterial properties of various phytochemicals has been undertaken to assess their potential utility in combatting infectious diseases [32]. The emergence of many drug-resistant human pathogens has spurred efforts to seek out new antimicrobial agents from natural sources [33]. Plants, in particular, are recognized for their ability to produce specific compounds with natural toxicity toward microorganisms, particularly those resistant to multiple drugs [34]. In the current investigation, the extracts derived from the investigated plant displayed different degrees of inhibitory potential against the tested microbes, as indicated in Figures 4 and 5. The results were quantified in terms of the clear zones representing the diameter of the growth inhibition area. The findings revealed the susceptibility of the tested microbes to ethanolic extract as indicated by the low MIC values against the microbes. The ethanolic crude extract of *P. hexandrum* exhibited the highest activity against *C. albicans* (27 ± 0.40 mm), followed by *E. coli* (19 mm ± 0.5), *S. aureus* (18 ± 0.27 mm), *E. faecalis* (16 ± 0.20 mm), and *P. aeruginosa* (15 ± 0.75 mm). However, the standard drug fluconazole did not show any effectiveness against *C. albicans*. A similar study on the ethanolic leaf extract of *P. hexandrum* reported strong antimicrobial potential against *B. subtilis, S. aureus, E coli,* and *P. aeruginosa* [35]. Another research conducted by Ahmad and Salam revealed that aqueous and methanolic rhizome extracts of *P. hexandrum* exhibited strong antimicrobial properties against *B. megaterium* and *P. aeruginosa*, resulting in inhibition zones ranging from 9 to 14 mm. Additionally, the extracts demonstrated inhibitory effects on fungi, specifically *A*. *flavus* and *F. solani*, with inhibition zones ranging from 9 to 18 mm [36]. Our results align with previous findings from various studies. In our research, the ethanolic extract displayed significant (*p* < 0.05) activity against all tested microorganisms, with MIC values ranging from 64 to 256 μg/mL. *C. albicans* showed the lowest MIC value of 64 μg/mL, followed by *E. coli* (128 μg/mL), *S. aureus* (128 μg/mL), *E. faecalis* (128 μg/mL), and *P. aeruginosa* (256 μg/mL). It is often reported in existing literature that the antibacterial characteristics found in higher plants are frequently attributed to secondary metabolites, such as phenolic, alkaloids, flavonoids, tannins, and other compounds [37]. Plant extracts exhibit antimicrobial activity through various mechanisms targeting essential microbial structures and functions. Bioactive compounds such as phenolics, flavonoids, and terpenoids disrupt microbial cell walls and membranes, increasing permeability and leading to cell lysis. These compounds also inhibit nucleic acid synthesis, protein translation, and enzyme activities crucial for microbial growth and survival. Additionally, they generate reactive oxygen species (ROS), causing oxidative stress that damages microbial DNA, proteins, and lipids. Some plant-derived compounds further interfere with biofilm formation and quorum sensing, weakening bacterial communication and resistance strategies. This multitarget approach makes plant extracts effective against diverse pathogens and promising alternatives in combating antibiotic resistance [38–40].

**Figure 6:**
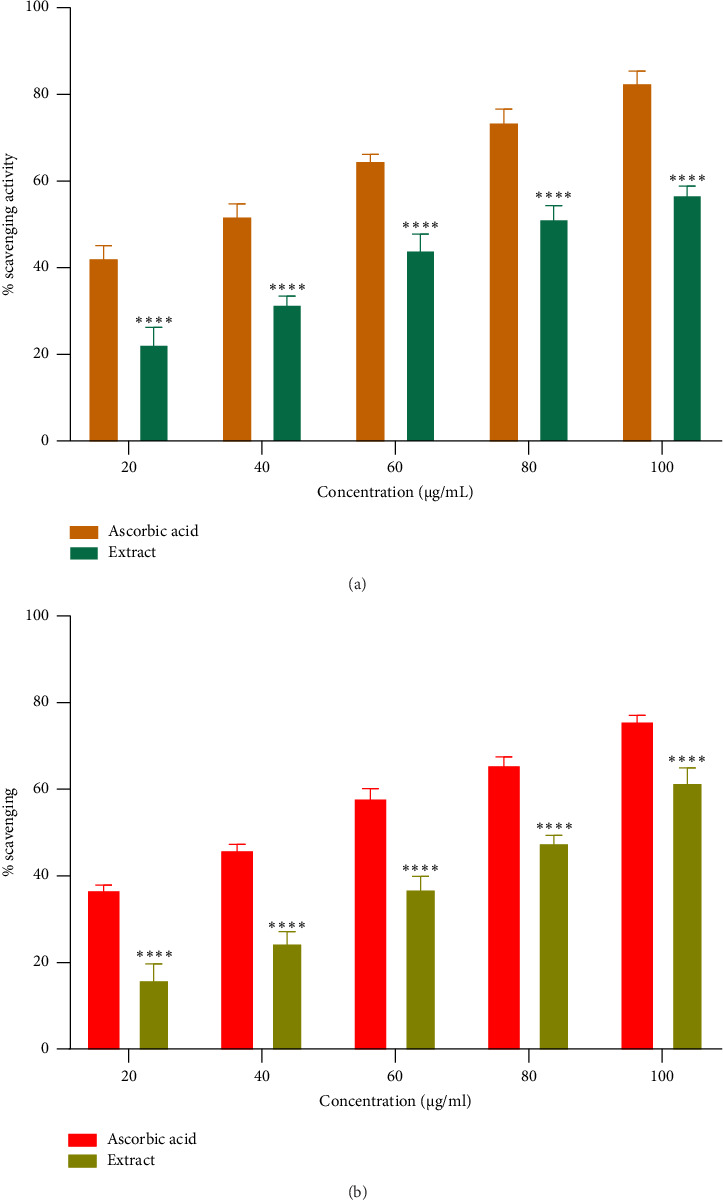
(a, b) DPPH and ABTS^+^ radical scavenging activity of *P. hexandrum* ethanolic extract and ascorbic acid at various concentrations (20–100 μg/mL). The graph illustrates a concentration-dependent increase in antioxidant activity for both the extract and ascorbic acid. The data are presented as mean ± S.D. from three independent experiments and analyzed by 2-way ANOVA followed by Sidak's multiple comparison test to determine the significance. Results indicated a significant difference between the standard and extract groups (^∗∗∗∗^*p* < 0.0001).

Free radicals are implicated in various diseases, including cancer and cardiovascular and neurodegenerative disorders. The ability of antioxidants to scavenge these radicals plays a crucial role in managing these conditions [41]. The DPPH and ABTS + assays are the most commonly employed methods for assessing the antioxidant potential of extracts from plants, fungi, or algae [42, 43]. The ethanolic root extract of *P. hexandrum* demonstrated concentration-dependent radical scavenging activity (Figures 6(a) and 6(b)). The highest DPPH scavenging activity was 59.23% at 100 μg/mL, with an IC_50_ value of 75.50 μg/mL. Similarly, for ABTS^+^, it was 61.81% at 100 μg/mL with IC_50_ value of 81.72 μg/mL. The results from this study are especially significant (*p* < 0.0001) when compared to previous research on *P. hexandrum*. In a previous study, the methanolic and ethanolic seed extracts of *P. hexandrum*, tested at a concentration of 700 μg/mL, showed DPPH scavenging activities of 86 ± 1.5% and 78 ± 1.2%, respectively [41]. Our results suggest that extract derived from the roots of *P. hexandrum* has the potential to protect against or prevent oxidative damage to fats, DNA, and proteins caused by ROS.

**Figure 7:**
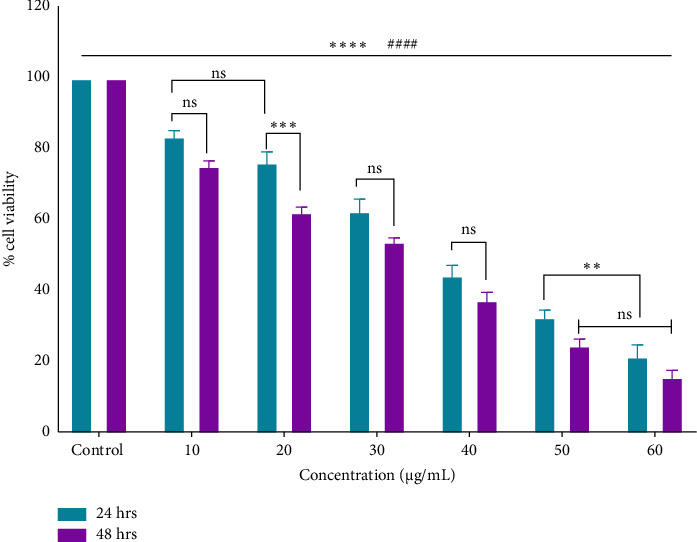
MTT assay showing the percentage of cell viability of HT-29 cells treated with different concentrations (10–60 μg/mL) of the *P. hexandrum* root extract for 24 and 48 hours. A significant decrease in cell viability is observed in a dose- and time-dependent manner. Data are expressed as the mean ± standard deviation from three independent experiments. Statistical significance is indicated as ⁣^∗∗∗∗^*p* < 0.0001, ^####^*p* < 0.0001 (comparison within 24 h and 48 h), ⁣^∗∗∗^*p* < 0.001, ⁣^∗∗^*p* < 0.01, and ‘ns' indicates no significant difference.

**Figure 8:**
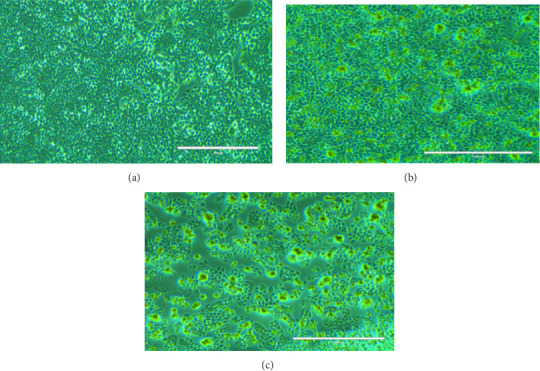
Morphological changes in HT-29 cells after treatment with *P. hexandrum* root extract, observed under a phase-contrast microscope. (a) Control cells; (b) cells treated for 24 hours at IC_50_ (38.7 μg/mL); (c) cells treated for 48 hours at IC_50_ (32.6 μg/mL). The number of cells decreased after the treatment and exhibited cell shrinkage and cytoplasmic membrane blebbing.

The research for innovative cancer treatments that do not cause side effects is rooted in ancient wisdom, particularly the use of traditional or ethnomedicinal plants. Many of these plants, such as those from the *Berberidaceae* family, including the *Podophyllum* genus, have long been recognized for their antiviral, anti-inflammatory, antidiabetic, antimicrobial, antioxidant, and anticancer effects [44, 45]. The current research observed the potent antiproliferative effects of an ethanolic root extract of *P. hexandrum* species native to the Kashmir Himalayas, India, on HT-29 cells. The findings of the MTT assay demonstrated that the ethanolic extract significantly (*p* < 0.0001) inhibited the proliferation of HT-29 cancer cells, with an IC_50_ of 38.7 μg/mL for 24 h and 32.6 μg/mL for 48 h. The MTT assay demonstrated that the ethanolic extract exhibited a dose-dependent inhibition of HT-29 cancer cell growth (Figures 7 and 8). These results indicate a promising potential for the ethanolic extract of *P. hexandrum* roots as an anticolon cancer agent.

After 24 h, the cell morphological changes occur, indicating a potential cytotoxic effect. This effect becomes more pronounced at 48 h, suggesting a time-dependent response to the treatment. In related studies, Zarger et al. [44] showed that seed extracts from *P. hexandrum* have strong inhibitory effects on MCF and HeLa cancer cell lines. The methanolic extract showed the highest inhibition, with 78% for MCF and 52% for HeLa, followed by the ethanolic extract, which showed 63% inhibition for MCF and 52% for HeLa at 100 μg/mL. Separately, Kitaeva et al. [46] found that extracts from the roots and rhizomes of *P. peltatum* exhibited cytotoxic effects on HeLa cells, reducing their viability by 52% to 60%. The anticolon cancer potential of other medicinal herbs used historically in the Kashmir Himalayas has also been studied. Extracts from *Rhodiola imbricata* suppressed the proliferation of HT-29 cancer cells, with acetone and methanol extracts at 200 μg/mL achieving an 84% reduction in cell growth [47]. Additionally, protease inhibitors from *Lavatera cashmeriana* seeds showed significant anticancer activity against Colo 205 and HCT-116 colon cancer cell lines, with IC_50_ values of 60 and 43 μg/mL, respectively [48]. In contrast to these plants, our findings showed a 50% inhibition of HT-29 cells at 38.20 μg/mL for 24 h and 32.5 μg/mL for 48 h, indicating that the ethanolic root extract of *P. hexandrum* has promising potential for combating colon cancer. It is important to note that while podophyllotoxin and its derivatives are well-known bioactive compounds in *P. hexandrum* responsible for its antitumor activity [15], these compounds were not detected in our GC-MS analysis, potentially due to geographical and environmental variations, seasonal differences, or extraction and analytical limitations [49–52]. However, the cytotoxic effects observed in our study suggest the presence of other bioactive compounds that may contribute to the anticancer activity of the extract. Despite the absence of podophyllotoxin, the potential toxicity of *P. hexandrum* extracts remains a critical consideration, as previous studies have shown that podophyllotoxin and related lignans can cause severe side effects, including myelosuppression, gastrointestinal toxicity, and hepatotoxicity, due to their mechanism of action involving microtubule disruption and topoisomerase II inhibition [53]. The observed cytotoxicity underscores the need for careful evaluation of the extract's safety profile. Future research should focus on identifying the specific bioactive compounds responsible for the cytotoxic effects, conducting toxicological evaluations to ensure safety for human consumption, and exploring advanced drug delivery systems (e.g., nanoparticles and liposomes) and combination therapies to enhance selective targeting of cancer cells and minimize off-target effects. Further studies are also needed to confirm the anticancer properties of *P. hexandrum* and to explore the mechanisms behind its effects.

## 4. Conclusion

In conclusion, the present study underscores the immense therapeutic potential of *P. hexandrum* root extract, enriched with diverse bioactive compounds. Its significant antimicrobial, antioxidant, and anticancer properties align with the historical use of medicinal plants in addressing complex health challenges. The observed antimicrobial activity against a broad spectrum of microbes, including *C. albicans* followed by *E. coli S. aureus, E. faecalis*, and *P. aeruginosa* coupled with potent antioxidant capabilities, highlights the extract's ability to mitigate oxidative stress and combat pathogenic organisms. Moreover, the pronounced cytotoxic effects on HT-29 cancer cells point to its promise as a natural anticancer agent. However, translating these findings into clinical applications requires further exploration, including detailed mechanistic studies, in vivo validations, and toxicity profiling. This study contributes to the ongoing efforts to harness plant-derived compounds as sustainable and effective solutions for modern healthcare challenges.

## Figures and Tables

**Figure 3:**
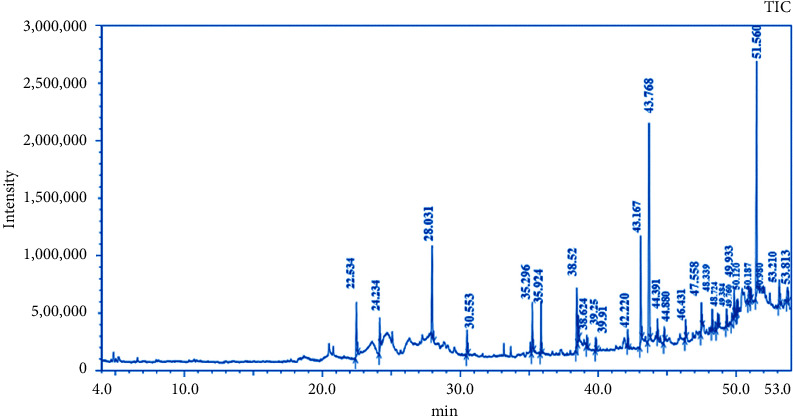
Chromatogram of the ethanolic root extract from *P. hexandrum*.

**Table 1:** The yield of extraction of *P. hexandrum* root in ethanol.

Species	Mass of the dried root (g)	Mass of the extract (g) yield (%)
*P. hexandrum*	20	2.2 (11%)

**Table 2:** Summary of the presence of various phytochemical compounds in the ethanolic extract of *P. hexandrum*.

Chemical test	Observation
Phenolic compound	(+)
Alkaloids	(+)
Flavonoids	(+)
Terpenoids	(+)
Saponins	(+)
Tannins	(+)
Steroids	(+)

*Note:* (+) result indicates the presence of the respective compound.

**Table 3:** List of the compounds characterized through GC-MS analysis in the ethanolic root extract of *P. hexandrum*.

Peak#	R. time	Area (%)	Name
1	22.534	4.13	4-Hydroxybenzene ethanol
2	24.234	2.14	Apocynin
3	28.031	6.66	α-Benzylphenethylamine
4	30.553	1.80	(E)-4-(3-Hydroxyprop-1-en-1-yl)-2-methoxyphenol
5	35.296	3.42	n-Hexadecanoic acid
6	35.924	3.11	Benzenepropanoic acid, 3,5-bis (1,1-dimethylethyl)-4-hydroxy-, methyl ester
7	38.527	5.45	9,12-Octadecadienoic acid (Z,Z)-
8	38.624	2.11	9,12,15-Octadecatrienoic acid, (Z,Z,Z)-
9	39.255	0.69	Phenol, 4,4′-(1-methylethylidene) bis-
10	39.910	1.04	Octadecamethyl-cyclononasiloxane
11	42.220	0.96	Cyclodecasiloxane, eicosamethyl
12	43.167	8.08	Trioxsalen
13	43.768	22.69	1,3,5-Triphenylcyclohexane
14	44.391	1.05	Eicosamethyl-cyclodecasiloxane
15	44.880	0.92	Hexadecanoic acid, 2-hydroxy-1-(hydroxymethyl)ethyl ester
16	46.431	1.20	Tetracosamethyl-cyclododecasiloxane
17	47.558	1.88	n-Propyl 9,12-octadecadienoate
18	48.339	1.39	Tetracosamethyl-cyclododecasiloxane
19	48.724	1.00	7H-Furo [3′,2′:4,5] furo [2,3-c] xanthen-7-one, 3a,12c-dihydro-8-hydroxy-6,10,11-trimethoxy-, (3aR,12cS)-
20	49.384	1.09	21H-Biline-1,3,19 (2H)-trione,17-ethyl-23,24-dihydro-2,2,7,8,12,13,18-heptamethyl-
21	49.760	0.69	Neotigogenin
22	49.933	1.76	Dibenzo [c,E]cycloheptene, 2,3,4,7-tetrameth
23	50.120	0.81	Tetracosamethyl-cyclododecasiloxane
24	50.187	0.85	5-(3,4,5-Trimethoxyphenyl)-2H-naphtho [2,3-d] [1,3] dioxolec-chloro-n-[2-(6,7-dimethoxy-isoq)]
25	50.980	1.79	1-Chloro-N-[2-[(6,7-dimethoxyisoquinolin-1-yl)methyl]-4,5-dimethoxyphenyl]methanesulfonamide
26	51.133	0.75	(.+−.)-5,12b-Dihydro-9,10-dimethoxy-1,3-dioxolo [4,5-g]isoindolo [1,2-a] isoquinolin-8 (6H)-one
27	51.560	18.70	2,2′-Benzylidenebis (3-methylbenzofuran)
28	53.210	2.2	511H-Cyclopenta [a]phenanthrene-15-carboxylic acid, 12.13,16,17-tetrahydro-3-methoxy-13-methyl-17-oxo-, methyl ester, (S)-
29	53.813	1.58	3,4-Bis [(trimethylsilyl)oxy] dihydro-2 (3H)-furanone

		100.00	

## Data Availability

All relevant data for this research are provided in the manuscript.
